# Post-partum pituitary insufficiency and livedo reticularis presenting a diagnostic challenge in a resource limited setting in Tanzania: a case report, clinical discussion and brief review of existing literature

**DOI:** 10.1186/1472-6823-12-4

**Published:** 2012-07-10

**Authors:** Faheem G Sheriff, William P Howlett, Kajiru G Kilonzo

**Affiliations:** 1 Kilimanjaro Christian Medical Centre, Moshi, Tanzania

**Keywords:** Post partum panhypopituitarism, Lymphocytic hypophysitis, Sheehan’s syndrome, Livedo reticularis, Africa

## Abstract

**Background:**

Pituitary disorders following pregnancy are an important yet under reported clinical entity in the developing world. Conversely, post partum panhypopituitarism has a more devastating impact on women in such settings due to high fertility rates, poor obstetric care and scarcity of diagnostic and therapeutic resources available.

**Case presentation:**

A 37 year old African female presented ten years post partum with features of multiple endocrine deficiencies including hypothyroidism, hypoadrenalism, lactation failure and secondary amenorrhea. In addition she had clinical features of an underlying autoimmune condition. These included a history of post-partum thyroiditis, alopecia areata, livedo reticularis and deranged coagulation indices. A remarkable clinical response followed appropriate hormone replacement therapy including steroids. This constellation has never been reported before; we therefore present an interesting clinical discussion including a brief review of existing literature.

**Conclusion:**

Post partum pituitary insufficiency is an under-reported condition of immense clinical importance especially in the developing world. A high clinical index of suspicion is vital to ensure an early and correct diagnosis which will have a direct bearing on management and patient outcome.

## Background

The pituitary gland undergoes major anatomic, physiologic and immunologic changes during pregnancy. Its enlargement is chiefly attributed to lactotroph hyperplasia [1]. These changes then predispose the pregnant woman to a spectrum of pituitary disorders in the intra-partum and post-partum periods. These include Sheehan’s syndrome which is by far the commonest, lymphocytic hypophysitis and rarely, apoplexy of pituitary adenomas [1,2].

Sheehan’s syndrome (SS) is becoming increasingly rare in the developed world due to improved standards of obstetric care; the same is not yet true for the developing world. The prevalence of women of reproductive age with suspected SS in the Kashmir valley (Indian subcontinent) was estimated at 3.2% [3]. Similar cross-sectional studies are virtually non-existent for Africa but a couple of case series have appeared in the literature. Cénac et al reported 40 cases of SS within a 5-year period at a hospital in Niger. All their patients were black African women living in rural areas and had no medical assistance during the last delivery [4]. Another group of researchers from Senegal noted that the main risk factors were traditions of home delivery and lack of obstetric care [5]. In addition, they observed a long latency period before the disease manifestations became overt [5]. Not surprisingly, lymphocytic adenohypophysitis with an estimated annual incidence in the UK of one case per 9 million [6], is even less commonly reported in sub-Saharan Africa with only a couple of isolated case reports till date from South Africa [7,8].

## Case presentation

### History

ZM, a 37 year old multiparous woman from Northern Tanzania, presented with complaints of generalized body swelling associated with progressive weight gain for ten years. The onset coincided with her last child birth which was complicated by mild post-partum haemorrhage following which she failed to lactate. She also experienced cold intolerance, loss of libido and a complete cessation of her menses. Four years prior admission she had had a serious febrile illness following which she experienced a brief period of altered level of consciousness and transient aphasia; since then she noted slowing of speech. As part of her systems review, she reported an anterior neck swelling which increased during her last pregnancy then gradually subsided a few months later. She also reported headaches of moderate intensity but no gross visual changes; she suffered from occasional rashes in sunlight exposed areas but no mucosal ulceration. Her past medical history revealed no previously diagnosed chronic illnesses; she was sero-negative for HIV and syphilis. Her obstetric history revealed she had a parity of five with four living children (one still birth at term with no obvious congenital malformations noted). Since she had received no formal antenatal care, there were no records of blood pressure measurements during pregnancy. All were home deliveries in the absence of a qualified birth attendant.

### Physical examination

**Vitals:** Pulse: 70/min; BP: 102/68 mm Hg; Temp: 36.2 degrees C; Respiratory rate 14/min. Orthostatics: Supine BP 110/80 mm Hg, standing BP 95/60 mm Hg. **General/ endocrine:** overweight woman (BMI 29. 1 kg/m^2^), looking younger than her stated age. She was pale with generalized, non-pitting edema involving face and extremities. Of note she had no oral ulcers and her thyroid gland was not palpable. She had “alabaster” skin with patchy hair loss over scalp; sparse axillary and pubic hair**. Cardiovascular:** Regular rate and rhythm with distant heart sounds. **Respiratory/Abdominal exams** were unremarkable**. Neurologic Exam:***Higher centres*: Fully oriented to time, place and person but with marked slowing of speech and mentation. *Cranial nerves*: Optic nerve- Visual acuity 20/30 both eyes; gross visual fields normal at bedside. Fundoscopy: no papilledema noted. Extra-ocular movements were intact. *Motor*: Limbs hypotonic; power reduced to grade 4/5 MRC (Medical Research Council grade) with proximal weaker than distal muscle groups. *Reflexes*: Ankle jerks were delayed and plantar responses flexor. *Sensation* was normal for all modalities tested.

### Investigations

#### Labs

Please see Table 1.

**Table 1:** Relevant laboratory results

CBC/ESR	Hb = 96.6 g/L (121–153), MCV = 83.3 fL (82–103), ESR = 60 mm/hr (0–10)
Coagulation panel	International Normalized Ratio (INR): 2.06 (1.0-1.5). The patient was not on any oral anti-coagulants; this had normalized on a follow-up exam. Activated Partial Thromboplastin Time(APTT): 31.15 sec (20–35)
Chemistry panel	Fasting blood glucose: 3.2 mmol/L (3.6 -6.3), Na^+^: 113.9 mmol/L (137–147), K^+^: 3.4 mmol/L (3.4-5.3), aspartate aminotransferase (AST): 83.6 IU/L (11–47); creatinine: 67 mcmol/L (44–150)
Dipstick urinalysis	proteinuria 2+, specific gravity: 1.030 (1.010-1.025)
Endocrine panel	T4: 38 ng/mL (60–160), T3: 0.8 ng/mL (1.0-3.1), TSH: 0.0 uIU/ml (0.4-6.2); LH, FSH, ACTH, GH, prolactin and cortisol could not be tested due to lack of reagents
Autoimmune panel	ANA (anti-nuclear antibody) titres less than 1:40; Anti-cardiolipin and anti-B2-glycoprotein IgM / IgG were all within normal limits. Thyroid auto-antibodies and angiotensin converting enzyme (ACE) levels could not be tested.

#### Computerized visual perimetry

Minor field defects were noted in both temporal fields.

#### Radiology

**Figure 1:**
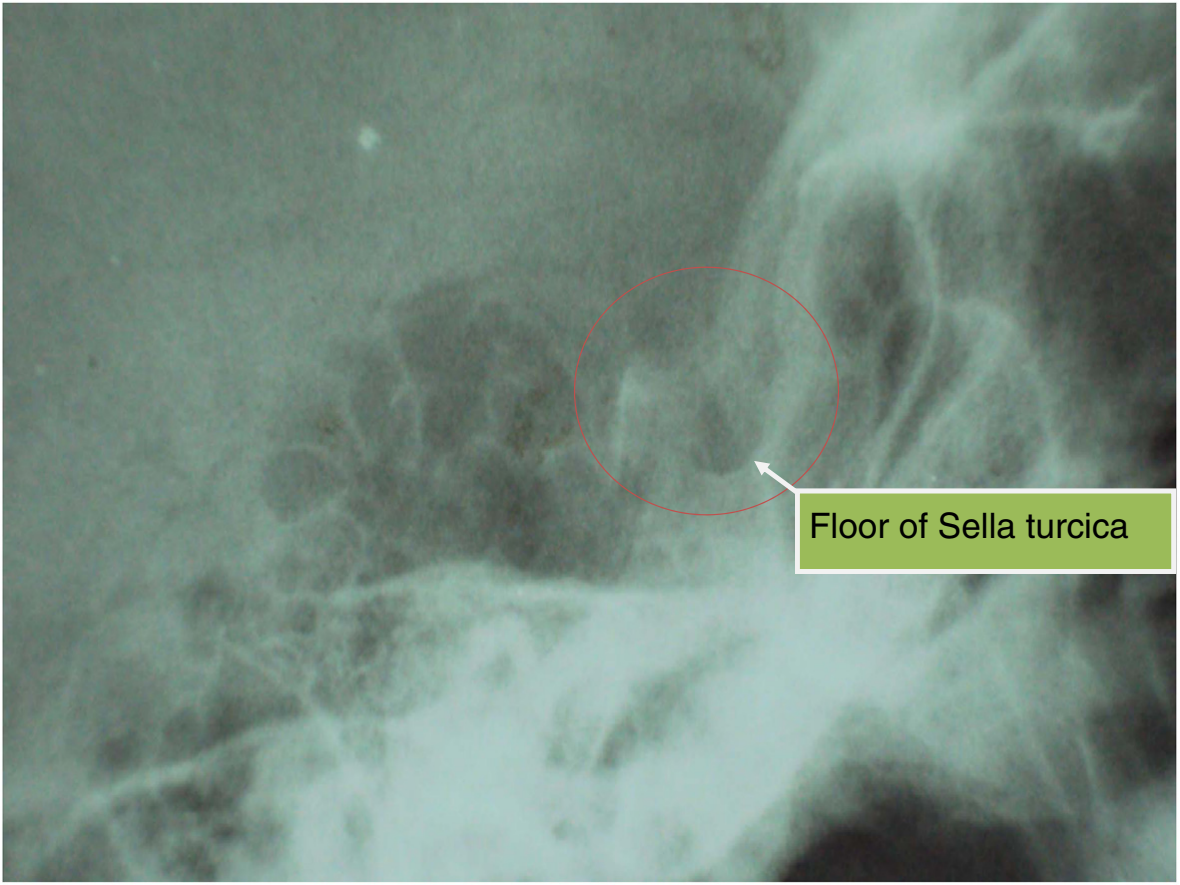
***Sella turcica X-ray*(lateral view).** Legend: No evidence of mass lesion seen, symmetric floor with no erosion or `double floor` sign.

**Figure 2:**
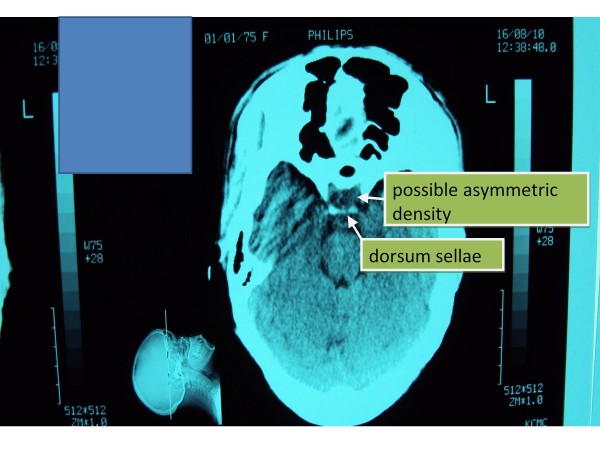
***Non-contrast axial head CT scan*(at the level of sella).** Legend: Possible asymmetric density within the pituitary gland on the left (but no ‘empty sella’ sign); dorsum sellae poorly visualized.

Chest X-ray: cardiomegaly, small left sided pleural effusion; Echo: 15 mm pericardial effusion, normal left ventricular function; X-ray of Sella: no evidence of mass lesion, symmetric floor (Figure 1); Non-contrast axial head CT scan: possible asymmetric density within the sella turcica (but no ‘empty sella’ sign); dorsum sellae poorly visualized (Figure 2).

### Management and progress in wards

**Figure 3:**
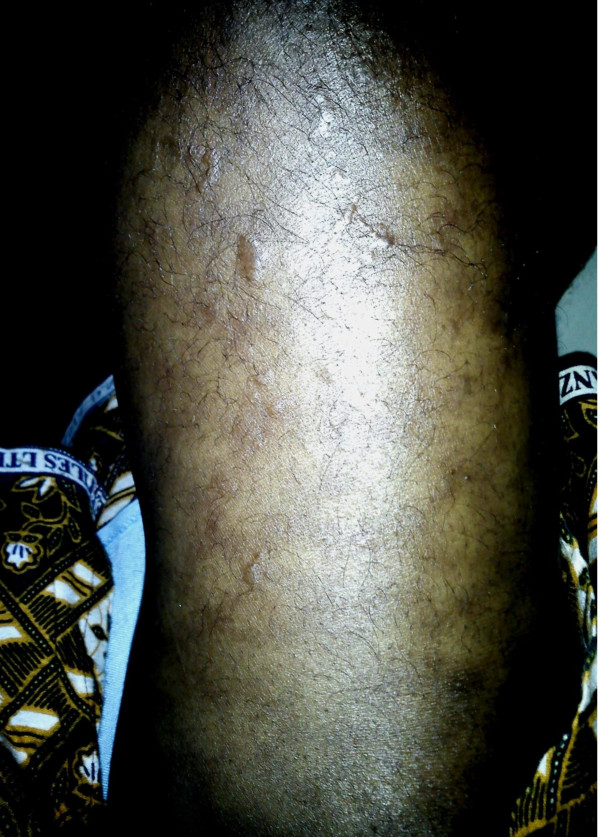
**Photograph of proximal left lower extremity at five-month follow up visit.** Legend: appearance of diffuse reticular rash consistent with livedo reticularis.

The patient was started on hormone replacement therapy including thyroxine 50 mcg daily, prednisone 5 mg AM and 2.5 mg PM and a combined oral contraceptive. During her stay in the wards the patient’s condition deteriorated abruptly due to an adrenal crisis probably precipitated by the vigorous thyroid hormone replacement. The dose of thyroxine was lowered to 25 mcg daily and the patient kept on normal saline and IV hydrocortisone 100 mg 6hourly. In addition, severe hyponatremia should be managed with water restriction and hypertonic saline infusion; the latter was avoided because of the difficulty monitoring Na^+^ levels and the associated risk of osmotic demyelination syndrome. Upon discharge three weeks later her rate of speech, mentation and exercise tolerance was significantly better compared to admission. On a two-month follow-up visit, there was a marked reduction in the generalised edema. The thyroxine dosage was subsequently increased gradually to 100 mcg daily. Her hair pattern had normalized in three months. At her five-month follow up visit, it was noted that she had developed livedo reticularis over her lower extremities bilaterally. (Figure 3) She was subsequently started on anti platelet therapy (junior aspirin 75 mg daily) and the oral contraceptive stopped. The livedo reticularis had disappeared on a subsequent visit. At 16 months, the patient was in good general health except for a headache and occasional palpitations. At this juncture, the thyroxine dose was lowered to 75 mcg daily.

### Differential diagnosis

This patient has anterior pituitary insufficiency beginning in the post partum period. Neurohypophyseal involvement is unlikely given the persistently elevated urine specific gravity even post steroid therapy which essentially rules out diabetes insipidus. The most likely differentials are lymphocytic hypophysitis (LyHy) and Sheehan’s syndrome. An underlying co-morbid auto-immune condition such as systemic lupus erythematosus (SLE) or anti-phospholipid syndrome is a relevant clinical consideration but is made less likely given her negative antibody screens. Neoplasms and other granulomatous disorders of the hypophysis (tuberculosis, syphilis, sarcoidosis and histiocytosis X) are also possibilities but would be lower on the list of differentials in the absence of appropriate clinical and laboratory evidence.

## Discussion

In this patient, the diagnosis of anterior pituitary insufficiency is evident from the constellation of secondary hypothyroidism, secondary amenorrhoea and the clinical manifestations of secondary adrenocortical insufficiency (hypoglycaemia, orthostatic hypotension and marked hyponatremia with low normal K^+^ levels). Interestingly, her TSH levels were undetectable which is quite rare in panhypopituitarism but has been previously reported [9]. Also noteworthy in this case is the severe hyponatremia which is likely multifactorial. In secondary adrenal insufficiency, one accepted explanation is that hypocortisolism leads to failure of inhibition of vasopressin secretion. In addition severe secondary hypothyroidism, which this patient also had, leads to a syndrome of inappropriate secretion of ADH (SIADH)-like picture [10-12].

Arriving at an etiologic diagnosis is more challenging. The history of post-partum hemorrhage albeit mild, and lactation failure may favor Sheehan’s syndrome (SS) over lymphocytic hypophysitis (LyHy) which is another recognized, but less common cause of post-partum pituitary insufficiency [1,2]. However in light of a history suggestive of painless post-partum thyroiditis, a physical exam which revealed signs of co-existing autoimmune conditions such as alopecia areata and the elevated ESR, the latter provides a better diagnostic fit [2,9,13,14]. While LyHy often presents with hyperprolactinemia from stalk dysfunction leading to galactorrhoea in a quarter to one-third of cases [1,6], agalactia has been reported in 11% of patients [6].

Although, thyroid auto-antibodies could not be tested and her antinuclear antibody and antiphospholipid antibodies were negative, a comprehensive retrospective analysis of 379 patients with lymphocytic hypophysitis (LyHy) by Caturgeli et al found that the prevalence of auto-antibodies for Hashimoto’s and SLE was only 7.4% and 1.3% respectively [6]. Similarly; anti-pituitary antibodies could not be assayed however these are considered of limited sensitivity and specificity in the diagnosis of lymphocytic hypophysitis since they are present in other autoimmune conditions and several non-immune pituitary disorders (including Sheehan’s syndrome) [6]. In the latter, post partum hemorrhage may trigger pituitary autoimmunity by the release of sequestered antigens following necrosis of the gland [15].

Assessment of visual fields is a simple but very useful diagnostic test. The minor defect in the patient’s temporal visual fields detected bilaterally upon visual perimetry may signify an ischemic process secondary to a mass effect at the optic chiasm – this could indicate a pituitary macroadenoma or an infiltrative process such as LyHy. The normal size sella on skull X-ray and CT scan might argue against a large tumor.

MRI is the imaging study of choice for the pituitary [16], the lack of which makes it difficult to accurately diagnose pituitary disease in most hospitals in the developing world. In any case, up to 9% of patients suffering from LyHy have normal imaging findings on CT/MRI. More common presentations include symmetric enlargement of sellar content (66%), thickening of the pituitary stalk (56%), homogenous enhancement (51%) and occasionally asymmetry of the enlarged sellar content (18%) [17]. This differs from the imaging findings in Sheehan’s syndrome which almost always results in a partially or completely empty sella that may be normal or reduced in size [18,19].

A definitive diagnosis usually requires a tissue biopsy often obtained via the endonasal transsphenoidal approach which was not possible in this case. Molitch et al have suggested criteria for making a strong presumptive clinical diagnosis non-invasively. These are as follows: a history of gestational / postpartum hypopituitarism, a contrast-enhancing sellar mass with MRI features characteristic of LyHy, a pattern of endocrine deficiency with early loss of adrenocorticotrophic hormone and thyroid-stimulating hormone unlike that found with macroadenomas and pituitary failure disproportionate to size of the mass [20].

Of note, livedo reticularis has never been reported before in patients with a clinical or histopathologic diagnosis of lymphocytic hypophysitis and may be unrelated. However, an association is not inconceivable particularly if the patient were to have an underlying co-morbidity such as SLE, since both LyHy and livedo reticularis have independently been documented in lupus [21,22]. This patient likely meets four (4) of the eleven (11) criteria required for a diagnosis of lupus namely photosensitivity, proteinuria, serositis (pleural and pericardial effusions) and hematologic abnormalities (normocytic anemia) [23]. However her ANA was negative and more specific tests such as anti-ds DNA or anti-Sm antibodies to rule out antinuclear-antibody negative disease were not available.

Finally, does a definitive diagnosis necessarily influence management in post-partum panhypopituitarism? The simple answer is yes - while hormone replacement is often all that is necessary for Sheehan’s syndrome, additional measures may be required for patients with LyHy presenting with symptoms of sellar compression. While surgery or pituitary radiotherapy may eventually be needed, in the absence of urgent visual symptoms it is reasonable to advocate using high dose glucocorticoids as first line therapy for LyHy under imaging surveillance if possible; decrease in volume of the pituitary mass and improving hormone status helps to confirm the diagnosis retrospectively [6,17]. Methotrexate and azathioprine can be used for poor responders [24-26]. Among the 320 patients with LyHy followed by Caturgeli, 73% required long term hormone replacement therapy, 16% recovered following mass-reduction without need for hormone-replacement, 8% died probably from irreversible adrenal insufficiency and 3% experienced spontaneous resolution without treatment [6].

## Conclusion

In the developing world, post-partum pituitary insufficiency is not altogether a rare clinical entity. The differential diagnosis becomes more challenging if clinical features suggestive of an auto-immune condition are also present. An accurate history and a keen physical exam become indispensable in correctly diagnosing and appropriately managing such patients especially in resource limited settings. A specific diagnosis may allow effective use of pharmacologic therapy before resorting to more invasive measures. There is a dire need for educating health care professionals and the general public since the disorder left undiagnosed has a devastating impact on maternal morbidity and mortality; properly designed epidemiologic studies to quantify the exact magnitude of the problem will be a necessary step towards the solution.

## Consent

The patient has provided explicit informed consent for publication of this case report and all accompanying images in a scientific journal.

## Abbreviations

ACTH: Adrenocorticotrophic hormone; ANA: Anti-nuclear antibody; BMI: Body mass index; ESR: Erythrocyte sedimentation rate; FSH: Follicle stimulating hormone; GH: Growth hormone; LH: Luteinizing hormone; LyHy: Lymphocytic hypophysitis; MCV: Mean corpuscular volume; SLE: Systemic lupus erythematosus; SS: Sheehan’s syndrome; TSH: Thyroid stimulating hormone.

## Competing interests

The authors declare that they have no competing interests.

## Authors’ contributions

FGS, WPH and KGK were involved in the initial case diagnosis. FGS and WPH were involved with revising the manuscript for publication. KGK continues clinical follow up of the patient to date. All authors read and approved the final manuscript.

## Pre-publication history

The pre-publication history for this paper can be accessed here:

http://www.biomedcentral.com/1472-6823/12/4/prepub

## References

[B1] Karaca Z Tanriverdi F Unluhizarci K Kelestimur F Pregnancy and pituitary disorders Eur J Endocrinol 2010 162 Suppl 3 453 475 19934270 10.1530/EJE-09-0923

[B2] Gutenberg A Hans V Maximilian J Primary hypophysitis: clinical-pathological correlations Eur J Endocrinol 2006 155 Suppl 1 101 107 16793955 10.1530/eje.1.02183

[B3] Zargar AH Singh B Laway BA Masoodi SR Wani AI Bashir MI Epidemiologic aspects of postpartum pituitary hypofunction (Sheehan's syndrome) Fertil Steril 2005 84 Suppl 2 523 528 16084902 10.1016/j.fertnstert.2005.02.022

[B4] Cénac A Soumana I Develoux M Touta A Bianchi G Sheehan's syndrome in Sudano-Sahelian Africa 40 observations Bull Soc Pathol Exot 1991 84 Suppl 5 686 692 1819419

[B5] Sidibe EH Sheehan’s syndrome: experience in Africa Ann Med Interne (Paris) 2000 151 Suppl 5 345 351 11033468

[B6] Caturegli P Newschaffer C Olivi A Pomper M Autoimmune hypophysitis Endocr Rev 2005 26 599 614 10.1210/er.2004-0011 15634713

[B7] Ouma JR Farrell VJ Lymphocytic infundibulo- neurohypophysitis with hypothalamic and optic pathway involvement: report of a case and review of the literature Surg Neurol 2002 57 Suppl 1 49 53 11834278 10.1016/s0090-3019(01)00647-4

[B8] Castle D De Villiers JC Melvill R Lymphocytic adenohypophysitis. Report of a case with demonstration of spontaneous tumour regression and a review of the literature Br J Neurosurg 1988 2 Suppl 3 401 405 3077051 10.3109/02688698809001013

[B9] Ozawa Y Shishiba Y Recovery from lymphocytic hypophysitis associated with painless thyroiditis: clinical implications of circulating antipituitary antibodies Acta Endocrinol 1993 128 493 498 8393255 10.1530/acta.0.1280493

[B10] Diederich S Franzen N Bahr V Oelkers W Severe hyponatremia due to hypopituitarism with adrenal insufficiency: report on 28 cases Eur J Endocrinol 2003 148 609 617 10.1530/eje.0.1480609 12773132

[B11] Raff H Glucocorticoid inhibition of neurohypophysial vasopressin secretion Am J Physiol 1987 252 635 644 10.1152/ajpregu.1987.252.4.R635 3032001

[B12] Erkut ZA Pool C Swaab DF Glucocorticoids suppress corticotropin-releasing hormone and vasopressin expression in human hypothalamic neurons J Clin Endocrinol Metab 1998 83 2066 2073 10.1210/jc.83.6.2066 9626140

[B13] Ajith C Gupta S Bhansali A Radotra B Kanwar A Kumar B Alopecia areata associated with idiopathic primary hypophysitis Clin Exp Dermatol 2005 30 250 252 10.1111/j.1365-2230.2005.01726.x 15807682

[B14] Honegger J Fahlbusch Bornemann Lymphocytic and granulomatous hypophysitis: experience with nine cases Neurosurgery 1997 40 Suppl 4 713 722 9092844 10.1097/00006123-199704000-00010

[B15] Goswami R Kochupillai N Crock PA Pituitary autoimmunity in patients with Sheehan's syndrome J Clin Endocrinol Metab 2002 87 Suppl 9 4137 4141 12213861 10.1210/jc.2001-020242

[B16] Errarhay S Kamaoui I Bouchikhi C Sheehan’s Syndrome: A case report and literature review http://www.ncbi.nlm.nih.gov/pmc/articles/PMC3066722/pdf/LJM-4-081.pdf 10.4176/081201 PMC3066722 21483515

[B17] Kristof RA Van Roost D Klingmüller D Lymphocytic hypophysitis: non-invasive diagnosis and treatment by high dose methylprednisolone pulse therapy? Neurol Neurosurg Psychiatry 1999 67 398 402 10.1136/jnnp.67.3.398 PMC1736542 10449568

[B18] Lee HC Lee EJ Lee KW Ahn KJ Jung TS Kim DI Huh KB Computed tomographic correlation with pituitary function in Sheehan's syndrome Korean J Intern Med 1992 7 Suppl 1 48 53 1477030 10.3904/kjim.1992.7.1.48 PMC4532096

[B19] Bakiri F Bendib SE Maoui R Bendib A Benmiloud M The sella turcica in Sheehan's syndrome: computerized tomographic study in 54 patients J Endocrinol Invest 1991 14 Suppl 3 193 196 1906495 10.1007/BF03346787

[B20] Molitch ME Gillam MP Lymphocytic Hypophysitis Horm Res 2007 68 Suppl 5 145 150 18174733 10.1159/000110611

[B21] Ji JD Lee SY Choi SJ Lymphocytic hypophysitis in a patient with systemic lupus erythematosus Clin Exp Rheumatol 2000 18 Suppl 1 78 80 10728449

[B22] Golden R Livedo reticularis in systemic lupus erythematosus Arch Dermatol 1963 87 299 301 10.1001/archderm.1963.01590150015002 13948706

[B23] Tan EM Cohen AS Fries JF Masi AT McShane DJ Rothfield NF The 1982 revised criteria for the classification of systemic lupus erythematosus Arthritis Rheum 1982 25 1271 1277 10.1002/art.1780251101 7138600

[B24] Leung GK Lopes MB Thorner MO Vance ML Laws ER Primary hypophysitis: a single-center experience in 16 cases J Neurosurg 2004 101 262 271 10.3171/jns.2004.101.2.0262 15309917

[B25] Tubridy N Saunders D Thom M Asa SL Powell M Plant GT Howard R Infundibulohypophysitis in a man presenting with diabetes insipidus and cavernous sinus involvement J Neurol Neurosurg Psychiatry 2001 71 798 801 10.1136/jnnp.71.6.798 11723207 PMC1737642

[B26] Lecube A Francisco G Rodriguez D Ortega A Codina A Hernandez C Simo R Lymphocytic hypophysitis successfully treated with azathioprine: first case report J Neurol Neurosurg Psychiatry 2003 74 1581 1583 10.1136/jnnp.74.11.1581 14617725 PMC1738221

